# Rapamycin Attenuated Zinc-Induced Tau Phosphorylation and Oxidative Stress in Rats: Involvement of Dual mTOR/p70S6K and Nrf2/HO-1 Pathways

**DOI:** 10.3389/fimmu.2022.782434

**Published:** 2022-02-07

**Authors:** Chencen Lai, Zhuyi Chen, Yuanting Ding, Qian Chen, Songbai Su, Heng Liu, Ruiqing Ni, Zhi Tang

**Affiliations:** ^1^Key Laboratory of Endemic and Ethnic Diseases, Ministry of Education & Key Laboratory of Medical Molecular Biology of Guizhou Province, Guizhou Medical University, Guiyang, China; ^2^Department of Nosocomial Infection, The First Affiliated Hospital of Guizhou University of Traditional Chinese Medicine, Guiyang, China; ^3^Department of Anesthesiology, Tongren Municipal People’s Hospital, Tongren, China; ^4^Institute for Regenerative Medicine, University of Zurich, Zurich, Switzerland; ^5^Institute for Biomedical Engineering, Eidgenössische Technische Hochschule Zürich (ETH) and University of Zurich, Zurich, Switzerland

**Keywords:** animal model, tau hyperphosphorylation, mTOR/p70S6K pathway, Nrf2/HO-1 (nuclear factor erythroid 2-related factor-2/heme oxygenase-1), oxidative stress, rapamycin, zinc, cognitive deficit

## Abstract

Alzheimer’s disease is pathologically characterized by abnormal accumulation of amyloid-beta plaques, neurofibrillary tangles, oxidative stress, neuroinflammation, and neurodegeneration. Metal dysregulation, including excessive zinc released by presynaptic neurons, plays an important role in tau pathology and oxidase activation. The activities of mammalian target of rapamycin (mTOR)/ribosomal S6 protein kinase (p70S6K) are elevated in the brains of patients with Alzheimer’s disease. Zinc induces tau hyperphosphorylation *via* mTOR/P70S6K activation *in vitro*. However, the involvement of the mTOR/P70S6K pathway in zinc-induced oxidative stress, tau degeneration, and synaptic and cognitive impairment has not been fully elucidated *in vivo*. Here, we assessed the effect of pathological zinc concentrations in SH-SY5Y cells by using biochemical assays and immunofluorescence staining. Rats (n = 18, male) were laterally ventricularly injected with zinc, treated with rapamycin (intraperitoneal injection) for 1 week, and assessed using the Morris water maze. Evaluation of oxidative stress, tau phosphorylation, and synaptic impairment was performed using the hippocampal tissue of the rats by biochemical assays and immunofluorescence staining. The results from the Morris water maze showed that the capacity of spatial memory was impaired in zinc-treated rats. Zinc sulfate significantly increased the levels of P-mTOR Ser2448, P-p70S6K Thr389, and P-tau Ser356 and decreased the levels of nuclear factor erythroid 2-related factor-2 (Nrf2) and heme oxygenase-1 (HO-1) in SH-SY5Y cells and in zinc-treated rats compared with the control groups. Increased expression of reactive oxygen species was observed in zinc sulfate-induced SH-SY5Y cells and in the hippocampus of zinc-injected rats. Rapamycin, an inhibitor of mTOR, rescued zinc-induced increases in mTOR/p70S6K activation, tau phosphorylation, and oxidative stress, and Nrf2/HO-1 inactivation, cognitive impairment, and synaptic impairment reduced the expression of synapse-related proteins in zinc-injected rats. In conclusion, our findings imply that rapamycin prevents zinc-induced cognitive impairment and protects neurons from tau pathology, oxidative stress, and synaptic impairment by decreasing mTOR/p70S6K hyperactivity and increasing Nrf2/HO-1 activity.

## Introduction

Alzheimer’s disease (AD) is pathologically characterized by abnormal accumulation of amyloid-beta (Aβ) plaques, neurofibrillary tangles (NFTs), neuroinflammation, oxidative stress, synaptic impairment, and neurodegeneration (1). Pathological changes lead to cognitive decline and impairment in patients and in animal models. The microtubule-associated protein tau is abnormally hyperphosphorylated and mainly aggregates into paired helical filaments (PHFs) in the brains of patients with AD (2, 3). Tau hyperphosphorylation is mediated by protein kinases or phosphatases, which are involved in AD neurofibrillary degeneration (4, 5). The mammalian target of rapamycin (mTOR) and ribosomal S6 protein kinase (p70S6K) are serine/threonine kinases that play key roles in the regulation of protein synthesis and degradation, age-dependent cognitive decline, and pathogenesis of AD (6–8). Accumulating evidence has demonstrated that abnormal mTOR signaling in the brain affects several pathways in AD that are associated with metabolism, insulin signaling, protein aggregation, mitochondrial function, and oxidative stress (9). Increased expression of mTOR and P70S6K colocalizes with NFT and mediates tau phosphorylation (6, 7, 10 8, 11). Rapamycin, a well-known inhibitor of mTOR, plays an important role in autophagy and insulin signaling (12, 13) and regulates tau phosphorylation (11, 14, 15). However, the upstream or downstream effectors controlled by mTOR that contribute to changes in neuronal functions and cognitive decline have not been fully elucidated.

Metal dysregulation, particularly iron, copper, and zinc, is implicated in the development of AD at an early stage (16–18 19–22). Higher levels of cerebral zinc are observed in postmortem brain tissue from patients with AD (reaching 200–300 μM) than in healthy controls (23, 24). Zinc is the second most abundant essential trace metal in the brain and is critical for maintaining brain homeostasis (22, 24, 25). Under pathological conditions, the excessive zinc released from synaptic vesicle activation promotes tau hyperphosphorylation (22, 23, 26, 27) in cells (11, 28) and liquid–liquid phase separation of tau protein (29). In addition, previous studies have demonstrated that zinc firmly binds to Aβ and was detected inside Aβ plaques (30–33). In the AD brain, presynaptic neurons release excessive zinc, which causes oxidase activation in neurons and exacerbates pathological development, leading to neuronal death (26, 27, 35, 38).

Oxidative stress is an early event in AD and plays an important role in AD pathogenesis (38). Elevated levels of reactive oxygen species (ROS) were detected in postmortem brain tissues from AD patients and animal models of AD (22, 36, 37). Activation of the nuclear factor erythroid 2-related factor-2 (Nrf2)/heme oxygenase-1 (HO-1) pathway inhibits the progression of inflammation and reduces ROS production and thus has been a potential therapeutic target for AD (39–42). There is a vicious cycle formed by excessive zinc, tau, and oxidative stress: elevated levels of zinc raise the production of ROS in mitochondria. Oxidative stress increases zinc concentration and tau hyperphosphorylation. In addition, excessive zinc and hyperphosphorylated tau cause oxidative stress and neurotoxicity. Hyperphosphorylated tau damages microtubule function and induces oxidative stress (43, 44). Increased oxidative stress has been indicated to cause tau hyperphosphorylation (45, 46) and aggravate neuronal death (47). Oxidative stress has been previously shown to be the underlying mechanism for the activation of mTOR in AD (48). However, the underlying mechanism of how excessive zinc links to tau degeneration remains unclear.

In the current study, we hypothesized that pathological concentrations of zinc could disturb the rapamycin-dependent mTOR/P70S6K and Nrf2/HO‐1 pathways, leading to detrimental effects on oxidative stress, tau hyperphosphorylation, and synaptic and cognitive impairment. To this end, we assessed the effect of rapamycin treatment on zinc sulfate (300 μM)-treated SH-SY5Y cells and lateral ventrally injected rats.

## Materials and Methods

### Materials and Antibodies

Zinc sulfate, rapamycin, trisaminomethane (Tris), radioimmunoprecipitation assay (RIPA), sodium dodecyl sulfate (SDS) buffer, and protease inhibitor cocktail were obtained from Sigma Aldrich Co. (St. Louis, MO, USA). A Bradford kit was purchased from Bio-Rad (CA, USA). For the primary antibodies employed in the present study, please refer to **Supplementary Table 1**.

### Cell Culture and Treatment

The cell culture was prepared as described previously (11, 49). Human SH-SY5Y neuroblastoma cells were grown to 70%–80% confluence in 75-cm^2^ plastic culture flasks (Corning, China) in a mixture of 5% CO_2_ and 95% air at 37°C, employing Dulbecco’s modified Eagle’s medium (DMEM)/F12 medium (1:1) supplemented with 10% fetal bovine serum (FBS), 100 units/ml penicillin, and 100 mg/ml streptomycin. Prior to treating SH-SY5Y cells with 300 μM zinc sulfate, the cultures were kept in free serum media. Zinc sulfate (300 μM) was chosen based on the results and protocol established in our previous study (49). SH-SY5Y neuroblastoma cells were pretreated with 20 ng/ml rapamycin for 1 h and then incubated with 300 μM zinc sulfate for 4 h.

For the generation of tau knockout cell line, the SH-SY5Y cells (5 × 10^5^) were seeded per well in a 6-well culture plate in a medium containing DMEM/F12, 10% FBS. The cells were grown until they reached 70%–80% confluence. We used SiRNA reagents (Invitrogen) to silence human Tau genes in the SH-SY5Y cells, following manufacturer’s instructions. The targeted RNA sequences that we used for Tau were: 5′CAUCCAUCAUAAACCAGGATT3′ (sense) and 5′UCCUGGUUUAUGAUGGAUGTT3′ (antisense).

### Animals

Eighteen Sprague–Dawley (SD) rats were included in this study (male, weight 250–300 g, 12 months of age, Guizhou Experimental Animal Center in China). All rats were housed in ventilated cages in a climate-controlled room (temperature: 22°C ± 2°C, humidity: 50% ± 5%, 12 h light–dark cycle with lights on at 8:00 a.m.). Food (safe, sterilized) and water (softened, sterilized) were provided *ad libitum*. Poplar wood shavings were placed in cages for environmental enrichment. All experimental protocols were approved by the Guiyang Regional Animal Care Center and Ethics Committee.

### Surgery and Treatment

**Figure 1:**
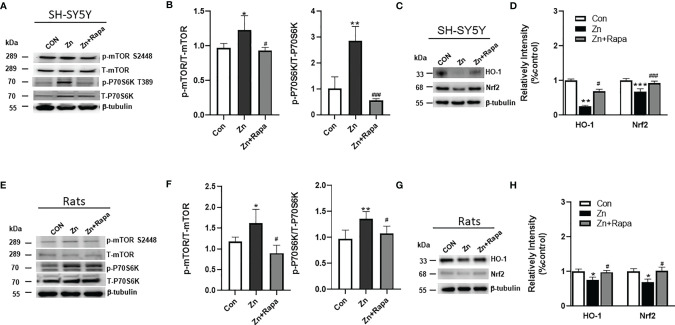
Effects of rapamycin on the expression of mammalian target of rapamycin (mTOR)/ribosomal S6 protein kinase (P70S6K) and nuclear factor erythroid 2-related factor-2 (Nrf2)/heme oxygenase-1 (HO-1) signaling pathways in zinc-treated SH-SY5Y cells and rats. **(A, B)** Representative blots and quantification of the expression ratio of p-mTOR S2448/T-mTOR and p-P70S6K T389/T-P70S6K in SH-SY5Y cells of the control, zinc, and zinc+rapamycin groups. n = 3 cell experiments per group. **(C, D)** Representative blots and quantification of the expression levels of HO-1 and Nrf2 were detected in cells of the control, zinc, and zinc+rapamycin groups. n = 3 cell experiments per group. **(E, F)** Representative blots and quantification of the expression ratio of p-mTOR S2448/T-mTOR and p-P70S6K T389/T-P70S6K in rats in the control, zinc, and zinc+rapamycin groups. n = 4 rats per group in the same membrane. **(G, H)** Representative blots and quantification of the expression levels of HO-1 and Nrf2 in rats in the control, zinc, and zinc+rapamycin groups. n = 4 rats per group in the same membrane. Quantifications of the blots were normalized to β-tubulin. *p < 0.05, **p < 0.01, ***p < 0.001 *vs.* control group; #p < 0.05, ###p < 0.001 *vs.* zinc+rapamycin treatment.

The timeline of the surgery, treatment, and behavior testing are shown in **Supplementary Figure 1**. All rats were randomly assigned into three groups (control group, zinc group, and zinc+rapamycin group; n = 6 each group). Rats were deeply anesthetized with an initial dose of 5% isoflurane in an oxygen/air mixture (1:4, 1 L/min) and were maintained at 1.5% isoflurane in an oxygen/air mixture (1:4, 0.6 L/min). Anesthetized rats were placed on a stereotaxic apparatus (RWD Life Science, Shenzhen, China), and the coordinates for injection were 0.8 mm posterior and 1.5 mm lateral and 3.6 mm ventral from bregma. Zinc sulfate (25 mM, 2 μl) was injected slowly into the right lateral ventricle in rats from both the Zn and Zn+rapamycin groups (**Figure 1A**). Rats in the control group underwent the same surgical procedures and were injected with phosphate-buffered saline (PBS; pH 7.4) of the same volume. The body temperature and respiratory rate of the rats were monitored during surgery. The body temperature of the animal was maintained at 36.5°C ± 0.5°C throughout the procedure using a warming pad. Lidocaine ointment was wiped locally to the scalp to reduce pain. One day after the surgery, rats in the zinc+rapamycin group were administered rapamycin (1.5 mg/kg body weight, intraperitoneal injection (i.p.), three times for 1 week, alternate day indicated in **Supplementary Figure 1**). The rats in the control and Zn groups were injected with 0.9% citrate buffer of the same volume (i.p.). Behavioral tests were subsequently performed after the rapamycin treatment period.

### Behavioral Testing

Morris water maze (MWM) was used to assess the hippocampal spatial learning function of the rats (50). The circular pool (160 cm diameter and 50 cm height) was filled to a depth of 30 cm with water (25°C ± 1°C) in this study. Visual cues were positioned above the water level, and extra maze cues were blocked with a dark curtain. During MWM training, all rats were subjected to 4 training trials daily for 6 consecutive days. In each trial, rats were trained to find a hidden platform (20-cm diameter) submerged 1 cm under the water surface for 60 s. Afterward, the rats were kept on the platform for 20 s. If these rats could not seek the platform within 60 s, they were guided to the platform within 60 s and kept on the platform for 20 s afterward. On day 7, a spatial probe trial was executed, where the platform was removed. The escape latency, the total time spent in the target quadrant, the number of platform crossings, and swimming speed were monitored by video tracking software (ANY-maze, USA).

After the behavioral tests, all rats were then sacrificed under deep anesthesia with pentobarbital sodium (50 mg/kg body weight) and transcardially perfused with PBS (pH 7.4). Brains were removed from the skull afterward. The left hemisphere brain tissue was saved for Western blot and stored at -80°C. The right hemisphere rat brain tissue was fixed in 4% paraformaldehyde in 1× PBS (pH 7.4) for 24 h and saved in 1× PBS (pH 7.4) at 4°C (51). For immunofluorescent staining, the fixed right brain hemisphere tissues were dehydrated using a vacuum infiltration processor (Leica ASP200S, Germany) and embedded in paraffin using an Arcadia H heated embedding workstation (Leica, Germany).

### Protein Extraction and Western Blotting

SH-SY5Y cells (n = 3 cell samples in each group) and the hippocampus of rats (n = 6 in each group) were lysed in RIPA buffer with a 0.1% protease inhibitor cocktail on ice. Protein concentration was measured by a Bradford kit (Bio-Rad). The proteins were analyzed by Western blotting as described earlier (11). The lysates were separated on a 7.5%–15% Sodium dodecyl sulfate polyacrylamide (SDS–PAGE) gel, and the bands were transferred onto 0.22/0.45-μm polyvinylidene difluoride (PVDF) membranes. After blocking the membranes with 5% milk, the membranes were incubated with primary antibodies (**Supplementary Table S1**) at 4°C overnight. The PVDF membranes were washed and then incubated with secondary peroxidase-coupled anti-mouse or anti-rabbit antibodies (1:5,000) at room temperature for 1 h (11). Immunoreactive bands were visualized by Immobilon Western horseradish peroxidase substrate luminol reagent (Millipore) using a ChemiDoc™ MP imaging system (Bio-Rad, USA).

### DCFH-DA Staining

ROS generation was measured *via* 2’-7’dichlorofluorescin diacetate (DCFH-DA) staining to detect intracellular hydrogen peroxide and oxidative stress (Beyotime, China). Following treatment and washing with PBS, the SH-SY5Y cells were incubated with DCFH-DA probes at 37°C for 30 min. The intracellular accumulation of fluorescent DCF was imaged using confocal microscopy (Leica, SP8, Germany). For each cell, the total area corresponding to DCF fluorescence was calculated. The experiments were repeated three times, and a total of 40–60 cells from each group were analyzed.

### Immunofluorescent Staining and Confocal Imaging

After treatment, SH-SY5Y cells were plated on coverslips, rinsed with PBS, and then fixed in 4% paraformaldehyde for 30 min. Cells were permeabilized in 0.1% Triton X-100 in Tris-buffered saline (TBS) for 10 min. The nonspecific binding sites were blocked with blocking solution (5% bovine serum albumin, 0.1% Triton X-100 in TBS) for 1 h. Cells were incubated with primary antibodies, anti-4-hydroxynonenal (4-HNE), anti-8-hydroxy-2’-deoxyguanosine (8-OHdG), and TOMM20, at 4°C overnight. After washing with TBS, bound antibody was detected by incubation for 1 h with Alexa Fluor 546-IgGs or Alexa Fluor 488-IgGs (1:200 for both, Invitrogen, USA). The fluorescence intensity was imaged using a Leica SP8 confocal microscope at 40× or 100× magnification. For each cell, the total area corresponding to 4-HNE- and 8-OHdG-related fluorescence was calculated. The experiments were repeated three times, and a total of 40–60 cells from each group were analyzed.

Coronal sections of the rat brains were cut at 6 μm using a microtome (Leica RM2245, Germany). Dewaxed and rehydrated hippocampal sections were blocked in TBST (TBS with Tween 20) with 5% bovine serum albumin for 1 h and then incubated with primary antibodies against 8-OHdG and NeuN at 4°C overnight. After washing, the sections were incubated with Alexa Fluor488 anti-mouse IgGs (1:200, Invitrogen, USA) for 1 h. After washing with TBS, the sections and coverslips were mounted with vector anti-fading mounting medium (Vector Laboratories, Burlingame, CA, USA). The fluorescence intensity was imaged using a Leica SP8 confocal microscope at 100× magnification. For each cell, the total area corresponding to 8-OHdG-related fluorescence was quantified using ImageJ 1.49 V software (NIH, USA). Neuronal Nuclei/4',6-diamidino-2-phenylindole (NeuN/DAPI) double-positive cells were counted and analyzed as a percentage of total DAPI+ cells. The experiments were from 6 sections, and a total of 30 cells from each group were analyzed.

### Cell Viability Assay

Cell viability was determined by Cell Counting Kit-8 (CCK-8, #G3581; Promega) according to the manufacturer’s instructions. Nearly 5 × 10^4^ cells were seeded into 96-well plates. After being treated, 20 μl of CCK-8 was added to each well, and then they were incubated for 4 h at 37°C. Finally, the absorption (450 nm) was measured using a Thermo Scientific Multiskan FC Microplate Reader.

### Statistical Analysis

Statistical analysis was executed using SPSS software (version 23.0) or GraphPad Prism 8.0 software. The results were presented as mean ± SEM. MWM behavior data were assessed by two-way repeated-measures ANOVA. The other parameters were executed using one-way ANOVA followed by Least significance difference (LSD)’s *post-hoc* test for multiple comparisons. Significance was set at p < 0.05.

## Results

### Downregulation of mTOR/P70S6K and Upregulation of Nrf2/HO‐1 Pathways Are Involved in the Protective Effects of Rapamycin Against the Toxic Effects of Zinc Sulfate Both in SH-SY5Y Cells and in Rats

First, we assessed the potential involvement of the mTOR/P70S6K pathway in zinc sulfate-induced alterations in SH-SY5Y cells and zinc sulfate-injected rats. Zinc sulfate significantly elevated the ratio of phosphorylated mTOR (S2448)/total mTOR by approximately 20% and phosphorylated P70S6K (T389)/total P70S6K in SH-SY5Y cells (p = 0.046 and p = 0.002, respectively) compared to the control group (**Figures 1A, B**). Pretreatment with rapamycin (20 ng/ml) abolished the effect of zinc sulfate on the phosphorylated levels of mTOR (S2448) and phosphorylated P70S6K (T389) in SH‐SY5Y cells, p = 0.029 and p = 0.001, respectively, in the zinc-treated group compared to the zinc+rapamycin-treated group (**Figures 1A, B**).

Zinc sulfate significantly elevated the ratio of phosphorylated mTOR (S2448)/total mTOR and phosphorylated P70S6K (T389)/total P70S6K by 44% and 38%, respectively, in the hippocampus of zinc-injected rats compared to control rats (p = 0.025 and p = 0.006, respectively) (**Figures 1E, F**). Rapamycin treatment attenuated the effect of zinc sulfate on mTOR (S2448) and phosphorylated P70S6K (T389) in rats, p = 0.02 and p = 0.029, respectively (zinc *vs.* zinc+rapamycin group) (**Figures 1E, F**).

Next, we assessed the potential involvement of the Nrf2 and HO‐1 pathways in zinc-induced alterations using SH-SY5Y cells and zinc-induced rats (**Figures 1C, D, G, H**). Zinc sulfate (300 mM) significantly reduced the levels of Nrf2 by approximately 70% and HO‐1 by 30% in SH‐SY5Y cells (p = 0.0003 and p = 0.016) (**Figures 1C, D**). Pretreatment with rapamycin (20 ng/ml) attenuated the effect of zinc sulfate on the levels of Nrf2 and HO‐1 (p = 0.001 and p = 0.043, respectively) in SH‐SY5Y cells treated with zinc compared to the zinc+rapamycin-treated group (**Figures 1C, D**).

In the hippocampus of zinc-injected rats, zinc sulfate (300 mM) significantly reduced the levels of Nrf2 and HO‐1 by approximately 20% and 30%, p = 0.031 and p = 0.04, respectively, zinc injected *vs.* zinc+rapamycin group (**Figures 1G, H**). Rapamycin treatment abolished the effect of zinc sulfate on the levels of Nrf2 and HO‐1 (p = 0.04 and p = 0.04, respectively) in rats in the zinc-injected *vs.* zinc+rapamycin group (**Figures 1G, H**). Hence, the neuroprotective effects of rapamycin were linked to inactivation of mTOR/P70S6K and activation of the Nrf2/HO‐1 signaling pathways as defensive responses to oxidative stress.

### Rapamycin Ameliorates Tau Hyperphosphorylation in Zinc-Induced SH-SY5Y Cells and Rats

**Figure 2:**
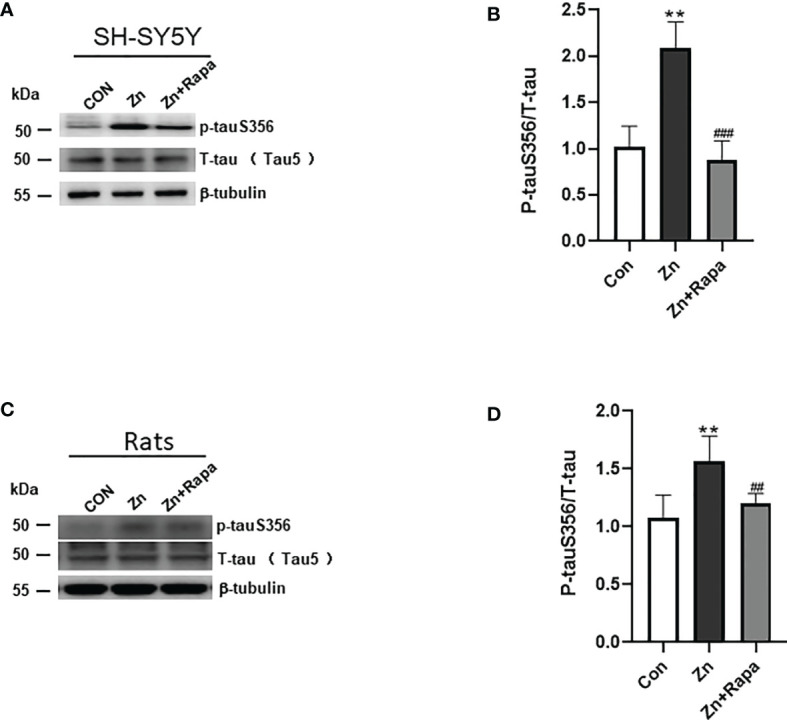
Rapamycin decreases the expression ratio of p-tau S356/T-tau in SH-SY5Y cells. **(A, B)** Representative blots and quantification of the expression ratio of p-Tau S356/T-tau (tau5) proteins in SH-SY5Y cells of the control, zinc, and zinc+rapamycin groups. n = 3 experiments per group. **(C, D)** Representative blots and quantification of the protein expression ratio of p-Tau S356/T-tau (tau5) in rats in the control, zinc, and zinc+rapamycin groups. n = 4 rats per group in the same membrane. **p < 0.01 *vs.* control group; ###p < 0.001 *vs.* rapamycin treatment.

Next, we assessed the potential rapamycin protection against zinc-induced tau hyperphosphorylation using SH-SY5Y cells and zinc-injected rats. We found that zinc treatment led to an increased ratio of hyperphosphorylated tau at Ser356 to total tau in zinc-treated SH-SY5Y cells compared to the control (p = 0.002). Rapamycin treatment completely restored the ratio of phosphorylated tau S356/total tau in SH-SY5Y cells, p = 0.001, zinc-treated compared to zinc+rapamycin-treated group (**Figures 2A, B**).

Moreover, zinc led to a 50% increase in the ratio of hyperphosphorylated tau at Ser356/total tau in the hippocampus of zinc-injected rats (p = 0.003) (**Figures 2C, D**). Rapamycin treatment decreased the ratio of phosphorylated tau S356/total tau by 30% in zinc-injected rats compared to the zinc+rapamycin group (p = 0.018). The total tau levels showed no change in the presence of zinc or zinc+rapamycin in rats (**Figures 2C, D**).

### Rapamycin Attenuates Oxidative Stress Damage in SH-SY5Y Cells and in the Hippocampus of Zinc-Induced Rats

**Figure 3:**
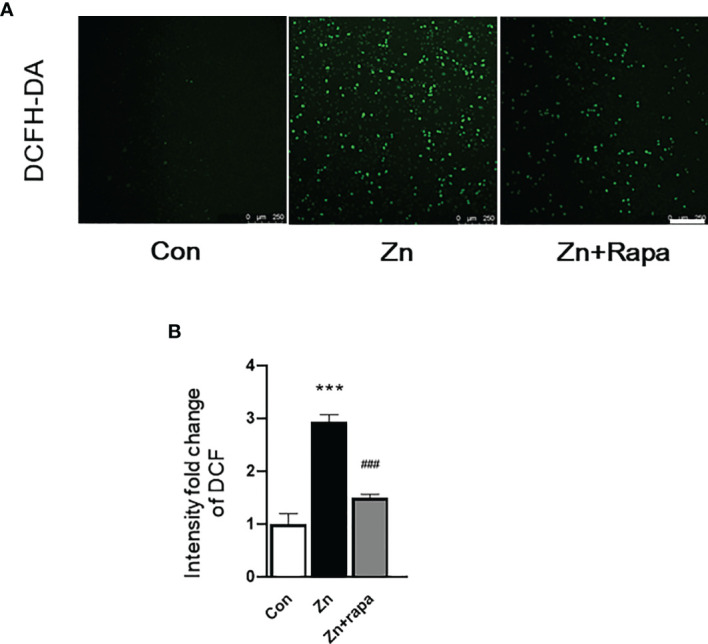
Rapamycin suppressed reactive oxygen species (ROS) generation caused by zinc in SH-SY5Y cells. **(A, B)** Representative fluorescent staining and quantification of the fluorescence intensity of ROS determined by 2’-7’dichlorofluorescin diacetate (DCFH-DA) staining in SH-SY5Y cells of the control, zinc, and zinc+rapamycin groups. Scale bar = 250 μm. n = 3 cell experiments per group, all confocal images represented as fold change relative to control cells. ***p < 0.001 *vs.* the control group; ###p < 0.001 *vs.* zinc+rapamycin treatment.

**Figure 4:**
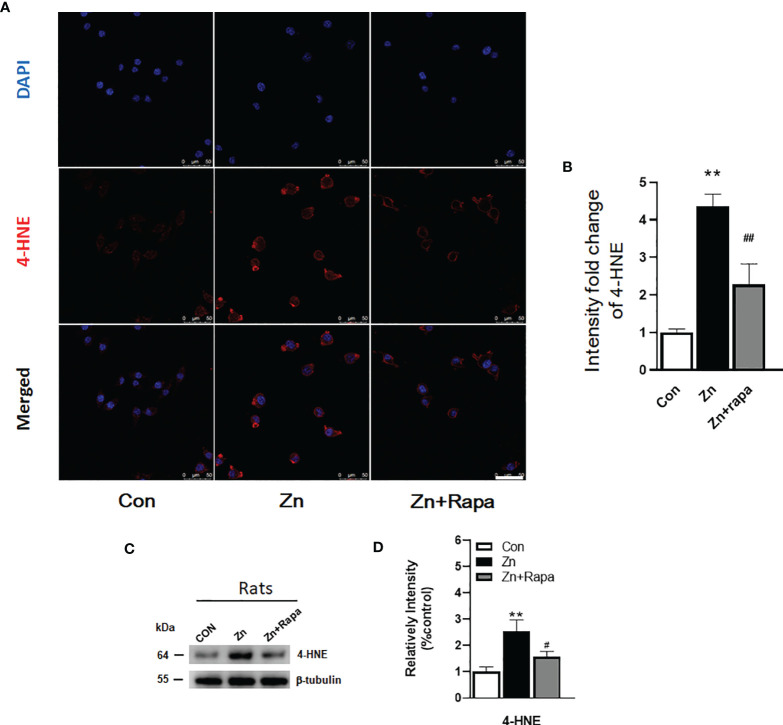
Rapamycin attenuated the zinc-induced increase in the expression of 4-hydroxynonenal (4-HNE) both *in vitro* and *in vivo*. **(A, B)** Representative confocal images and quantification of the fluorescence intensity for immunofluorescence staining using anti-4-HNE antibody (red) in SH-SY5Y cells in the control, zinc, and zinc+rapamycin groups, scale bar = 50 μm. Nuclei were counterstained with DAPI (blue). All confocal images are presented as the fold change relative to control cells. **(C, D)** Representative blots and quantification of the expression levels of 4-HNE in rats in the control, zinc, and zinc+rapamycin groups. n = 4 rats per group in the same membrane. **p < 0.01 *vs.* control group; #p < 0.05, ##p < 0.01 *vs.* rapamycin treatment. Quantifications of the blots were normalized to β-tubulin.

**Figure 5:**
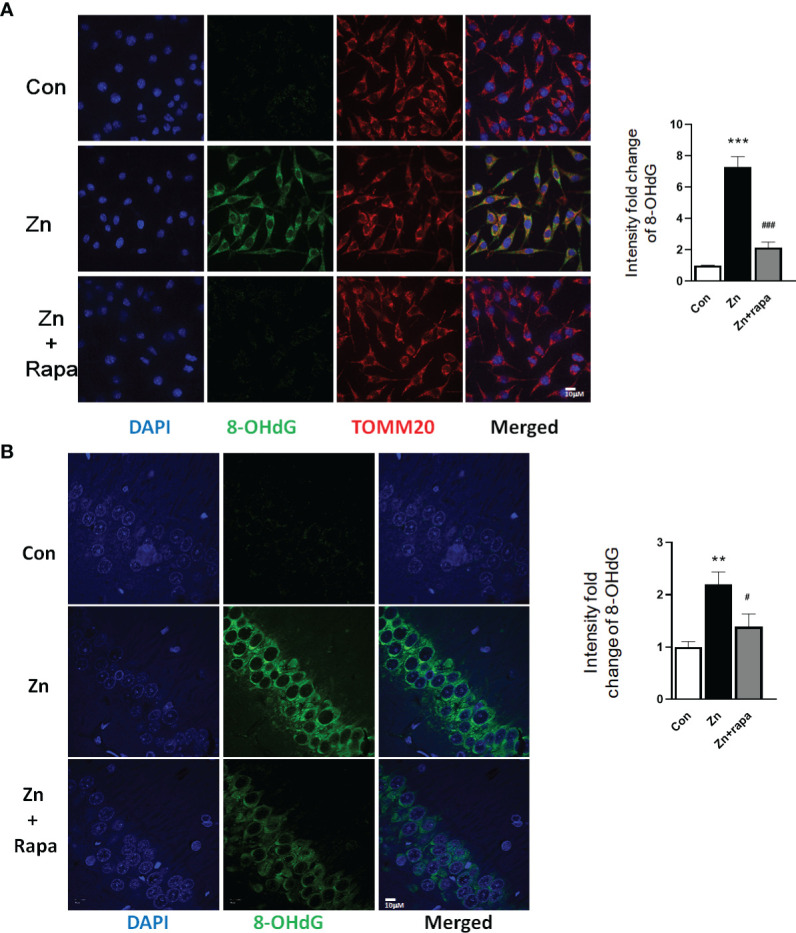
Rapamycin prevents the DNA oxidation caused by zinc in SH-SY5Y cells and rats. **(A, B)** Representative confocal images and quantification of the fluorescence intensity for immunofluorescence staining using anti-8-hydroxy-2’-deoxyguanosine (8-OHdG) antibody (green) and anti-TOMM20 (red) in SH-SY5Y cells in the control, zinc, and zinc+rapamycin groups. Scale bar = 10 μm; nuclei were counterstained with DAPI (blue). Quantification of the fluorescence intensity of 8-OHdG staining in the SH-SY5Y cells of the control, zinc, and zinc+rapamycin groups. n = 3 cell experiments per group. A total of 40–60 cells from each group were analyzed, and all confocal images are presented as the fold change relative to control cells. **(C, D)** Representative confocal images and quantification of the fluorescence intensity for immunofluorescence staining using anti-8-OHdG antibody (green) in the CA1 areas of the brains of the rats in the control, zinc, and zinc+rapamycin groups. Nuclei were counterstained with DAPI (blue). Scale bar = 10 μm; n = 6 rats per group. All confocal images represent the fold change relative to control rats. **p < 0.01, ***p < 0.001 *vs.* control group; #p < 0.05, ###p < 0.001 *vs.* zinc+rapamycin treatment.

Next, we assessed oxidative stress by immunofluorescence staining using DCFH-DA, 4‐HNE (lipid peroxidation), and 8‐OHdG (oxidation of DNA) in SH-SY5Y cells. Following exposure to zinc sulfate, the fluorescence intensities of DCFH-DA, 4‐HNE, and 8‐OHdG were elevated in the zinc-treated group compared to the control group in SH-SY5Y cells (p = 0.0001, p = 0.001, p = 0.0001, respectively) (**Figures 3A, B**; **4A, B**; **5A, B**). Prior treatment with rapamycin neutralized the zinc-induced increase in fluorescence intensity of DCFH-DA, 4‐HNE, and 8‐OHdG, zinc-treated group compared to zinc+rapamycin group in SH-SY5Y cells, p = 0.0004, p = 0.008, p = 0.0001, respectively (**Figures 3A, B**; **4A, B**; **5A, B**). Furthermore, the expression of 8-OHdG was predominantly localized to the cytoplasm and had a significant colocalization with a mitochondrial marker (anti-TOMM20).

To determine oxidative stress damage induced by zinc *in vivo* in rats, we measured the levels of 4-HNE in hippocampal brain tissue homogenates from rats by using Western blotting. Zinc led to an increased level of 4-HNE in the hippocampus of zinc-injected rats (p = 0.005) compared to the control group (**Figures 4C, D**). Rapamycin treatment decreased the level of 4-HNE in zinc-injected rats compared to the zinc+rapamycin group (p = 0.04) (**Figures 4C, D**). We further measured the levels of 8-OHdG products by immunofluorescence staining in hippocampal brain tissue slices from rats. Zinc led to an increased level of 8-OHdG in the hippocampus of zinc-injected rats (p = 0.001) compared to the control group (**Figures 5C, D**). Rapamycin treatment decreased the levels of 4-HNE and 8-OHdG in zinc-injected rats compared to the zinc+rapamycin group, %, p = 0.013 (**Figures 5C, D**).

### Rapamycin Rescued Impaired Learning and Memory in Zinc-Induced Rats

**Figure 6:**
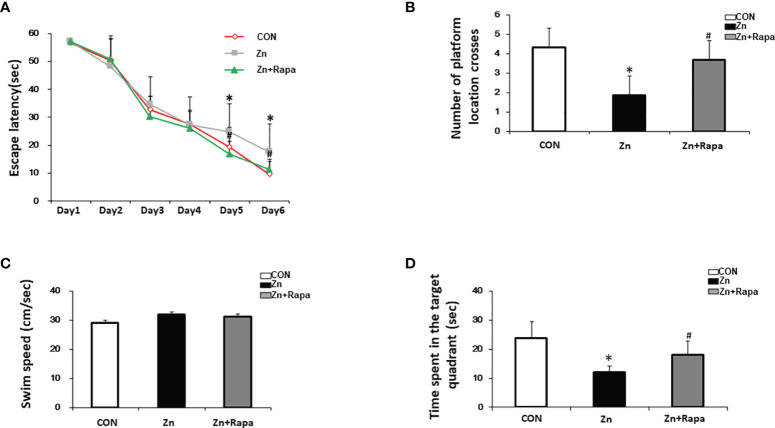
The effect of rapamycin on the performance of the Morris water maze (MWM) in zinc-induced rats. **(A)** Escape latency of the rats in all three groups [control (red), zinc-injected (gray), zinc+rapamycin treatment (green)] in the MWM test of each training day. **(B)** Representative number of platform crossings on day 7. **(C)** Representative time spent in the target quadrant on day 7. **(D)** Swimming speed in the MWM on day 7. n = 6 rats per group, *p < 0.05 *vs.* control group; #p < 0.05 *vs.* rapamycin treatment.

The spatial learning and memory function of the rats were assessed by using the MWM test. The escape latency significantly increased in the zinc-injected rats compared to the control on Day 5 and Day 6 (p = 0.031, p =0.024, respectively) (**Figure 6A**). On day 7, the time spent in the target quadrant and the number of platform location crossings both decreased by approximately 50% in zinc-induced rats compared to the control group (p = 0.027 and p = 0.039) (**Figures 6B, D**).

Treatment with rapamycin led to a reduced escape latency in zinc+rapamycin rats compared to zinc-injected rats on Day 5 and Day 6 (p = 0.021, p = 0.033, respectively). On day 7, the time spent in the target quadrant and the number of platform location crossings were increased compared to zinc-injected rats (p = 0.044, p = 0.044) (**Figures 6B, D**). To study whether zinc could affect the motion ability of rats, the swimming speed of rats was recorded. No differences were observed among the three groups (**Figure 6C**), implying that treatment with rapamycin and zinc did not radically affect the motion ability of rats.

### Rapamycin Protected Synapses in Zinc-Induced SH-SY5Y Cells and in Rats and Rescued Hippocampal Neuronal Death in Zinc-Induced Rats

**Figure 7:**
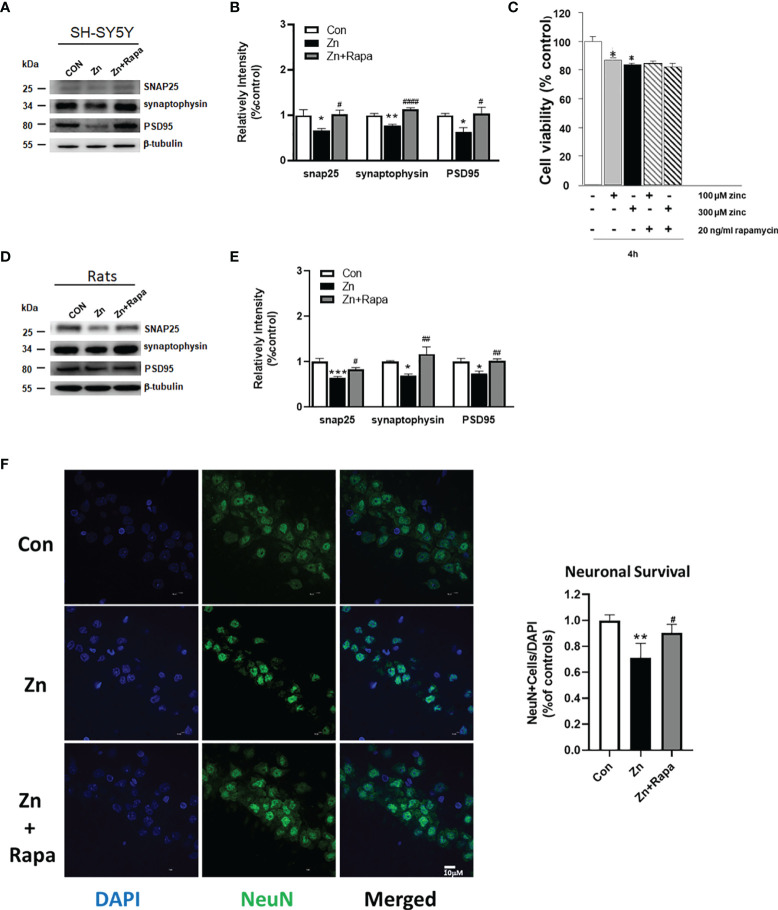
Rapamycin improved synaptic impairment caused by zinc in both SH-SY5Y cells and rats and partially rescued cell death in zinc-induced rats. **(A, B)** Representative blots and quantification of the protein expression levels of SNAP25, synaptophysin, and PSD-95 in SH-SY5Y cells in the control, zinc, and zinc+rapamycin groups. n = 3 cell experiments per group. **(C)** Cell viability was assessed using the Cell Counting Kit-8 (CCK‐8) assay. Cells were pretreated with rapamycin for 1 h and then treated with 100 or 300 µM zinc sulfate for an additional 4 h. **(D, E)** Representative blots and quantification of the protein expression levels of SNAP25, synaptophysin, and postsynaptic density protein-95 (PSD-95) in rats in the control, zinc, and zinc+rapamycin groups. n = 4 rats per group in the same membrane. **(F)** Representative NeuN immunostaining (green) image of the CA1 areas of the brains of the rats in the control, zinc, and zinc+rapamycin groups. DAPI staining showed the nuclei. NeuN/DAPI double-positive cells were counted and analyzed as a percentage of total DAPI+ cells. Scale bar = 10 μm; n = 6 rats per group, *p < 0.05, **p < 0.01, ***p < 0.001 *vs.* control group; #p < 0.05, ##p < 0.01, ####p < 0.0001 *vs.* zinc+rapamycin treatment. Quantifications of the blots were normalized to β-tubulin.

Next, we assessed the potential effect of rapamycin on zinc-induced synaptic impairment and cell death using SH-SY5Y cells and zinc-induced rats. The expression levels of presynaptic proteins [Synaptosomal-associated 25 kDa protein (SNAP) 25 and synaptophysin] and postsynaptic density protein-95 (PSD-95) are indicators of synaptic function. We found that zinc treatment led to reduced levels of SNAP 25, synaptophysin, and PSD-95 in SH-SY5Y cells treated with zinc compared to the control (p = 0.039, p = 0.004, and p = 0.04, respectively). Rapamycin treatment reversed the zinc-induced reduction in the levels of SNAP 25, synaptophysin, and PSD-95 in SH-SY5Y cells (p = 0.03, p = 0.0001, and p = 0.035) (zinc-treated group compared to zinc+rapamycin group) (**Figures 7A, B**). To assess cell death by performing CCK-8 assays, we found that at least 20% cell death was induced by 100 or 300 µM zinc sulfate for 4 h in SH-SY5Y cells, but rapamycin treatment did not affect cell death in SH-SY5Y cells (**Figure 7C**).

Moreover, zinc injection led to reduced levels of SNAP 25, synaptophysin, and PSD-95 in the hippocampus of zinc-injected rats (p = 0.001, p = 0.04, p = 0.013, respectively) (**Figures 7C, D**). Rapamycin treatment decreased the levels of SNAP 25, synaptophysin, and PSD-95 in zinc-injected rats compared to the zinc+rapamycin group (p = 0.038, p = 0.006, p = 0.009, respectively) (**Figures 7D, E**). NeuN (green) staining showed that zinc resulted in extensive neuronal loss in hippocampal CA1 neurons in zinc-treated rats compared with control rats (p = 0.0067), and rapamycin treatment partially rescued hippocampal CA1 neuronal death in rats (zinc-treated group compared to zinc+rapamycin group) (p = 0.038) (**Figure 7F**).

To determine the extent to which tau phosphorylation is related to the presynaptic impairment, we quantified the protein expression levels of SNAP25, synaptophysin, PSD-95, and Total-tau in SH-SY5Y cells in the control and Tau siRNA groups (tau knockout cell line). We showed that reduced tau is beneficial for maintaining synaptic function in SH-SY5Y cells to a certain extent (**Supplementary Figures 2A, B**).

## Discussion

Our data reveal that zinc leads to tau hyperphosphorylation, oxidative stress, and synaptic impairment involving mTOR/P70S6K activation and Nrf2/HO‐1 inactivation. Rapamycin ameliorates zinc-induced tau hyperphosphorylation, oxidative stress damage, and synaptic impairment and rescues spatial learning deficits by downregulating mTOR/P70S6K activities and upregulating Nrf2/HO‐1 activities.

Zinc has been closely related to cognitive function and plays an important role under physiological conditions. Elevated levels of zinc ions were found in AD brains, notably in the hippocampus, cortex, and amygdala, which are severely affected by NFTs (22, 52–54). Accumulating evidence has shown a tight relationship of zinc with tau degeneration and cognitive impairment in human patients (55). Dietary zinc supplementation treatment in 3xTg-AD mice has been shown to increase Brain-derived neurotrophic factor (BDNF) levels and prevent cognitive deficits and mitochondrial dysfunction (56). The pathological level of zinc promotes tau tangle pathology in the brains of hAPP/htau (57) and tau mouse models (58). Tau transgenic mice are featured by synaptic dysfunction, neuronal loss, neuroinflammation, and impairment in white matter integrity associated with tau accumulation (59–64). Abnormal accumulation of targeting metals has been shown to rescue the pathology and phenotype of transgenic mouse models of tauopathy (65).

In the present study, we found that excessive zinc could induce mTOR(S2448)-P70S6K(T389)-dependent phosphorylation and tau hyperphosphorylation in cultured neuroblastoma SH-SY5Y cells. Rapamycin suppresses mTOR (S2448)/P70S6K (T389) phosphorylation and ameliorates tau pathology. We found that lateral ventricular injection of zinc sulfate could induce persistent mTOR(S2448)-P70S6K(T389) phosphorylation in the rat hippocampus and that rapamycin reversed both mTOR(S2448) and P70S6K(T389) phosphorylation and reduced the level of tau hyperphosphorylation. These results implied that zinc played a crucial role in tau pathology by regulating the mTOR/P70S6K pathway and that rapamycin exerted a beneficial effect on tau pathology.

Zinc sulfate (300 uM) treatment has been shown inducing cell death. An et al. (28) showed that exposure to 100 μM zinc sulfate for 4 or 8 h caused a 20% reduction in SH-SY5Y cell viability. An et al. (28) and our previous studies have reported that zinc could induce increased phosphorylated p70S6K, p-PKB, and p-mTOR, and these kinases could induce tau phosphorylation (10, 11). Our results in the current study were in line with a previous finding: 300 μM zinc sulfate treatment induced more than 20% cell death (**Figure 7C**).

mTOR or P70S6K is one of the most important serine/threonine kinases in eukaryotic cells and plays a prominent role in the regulation of protein synthesis, phosphorylation, and autophagy (8, 66–70). We have previously shown increased expression of p-mTOR (S2448) and p-P70S6K (T389) in postmortem AD brains, which is associated with the accumulation of hyperphosphorylated tau in AD (10, 11, 71). This suggests that phosphorylation of mTOR at S2448 and P70S6K at T389 is a vital target for disease intervention. Zinc has been indicated to be involved in the mechanisms of mTOR/P70S6K activation in AD. We have previously shown that zinc treatment (300 μM) promoted tau phosphorylation *in vitro* in cell cultures (11). Another study demonstrated that synaptic zinc promoted tau hyperphosphorylation (27) and accelerated the fibrillization of mutant ΔK280 of full-length human tau, inducing apoptosis and toxicity in SH-SY5Y cells by bridging Cys-291 and Cys-322 (72). In addition, zinc binds to protein phosphatase 2A and induces its inactivation and tau hyperphosphorylation through Src-dependent PP2A (tyrosine 307) phosphorylation (73). In ApoE4 transgenic mice, tau hyperphosphorylation is associated with activation of extracellular signal-regulated kinase modulated by zinc (74).

Oxidative stress has been recognized as a causative factor in various neurodegenerative diseases (36, 37, 41, 42). Excessive zinc triggers oxidase activation and further generates oxidative products in neurons (22, 24). Pathological tau damages mitochondrial function, resulting in increased ROS products and causing oxidative stress (23, 24, 75, 76). Increased production of ROS causes lipid peroxidation and DNA damage and, in turn, could affect the hyperphosphorylation of tau, leading to a vicious cycle (37, 45, 76–78). Here, we investigated the mechanism of zinc-mediated tau pathology in AD and whether excessive zinc could exacerbate the vicious cycle through the mTOR/P70S6K pathway. The levels of oxidative products were assessed by immunostaining or Western blot in the present study. We found an increasing level of ROS accumulation in zinc-treated SH-SY5Y cells and markedly increased expression of lipid peroxidation product (4-HNE) and nucleic peroxidation product (8-OHdG) in both zinc-induced SH-SY5Y cells and zinc-injected rats. After rapamycin preadministration, the levels of ROS, 4-HNE, and 8-OHdG were decreased in zinc-treated SH-SY5Y cells and zinc-injected rats. Furthermore, we found that increased expression of 8-OHdG, a biomarker of DNA oxidative damage, was significantly colocalized with a mitochondrial marker (TOMM20) in zinc-treated SH-SY5Y cells. The increased expression of 8-OHdG was observed predominantly in the cytoplasm of SH-SY5Y cells, suggesting that zinc induced mitochondrial DNA (mtDNA) oxidative damage in SH-SY5Y cells. Mecocci et al. (79) reported that a small amount of DNA significantly increased oxidative damage to nuclear DNA (nDNA) and a highly significant 3-fold increase in oxidative damage to mtDNA in AD compared with age-matched controls. These findings indicate that zinc may induce mtDNA oxidative damage through the Nrf2/HO-1 pathway and that Nrf2/HO-1 pathway-associated ROS generation may play an essential role in the neurodegenerative process (79–82).

Nrf2 is a transcription factor that negatively regulates the level of ROS to protect against oxidative stress damage. Ramsey et al. (83) reported a significant decline in the level of Nrf2 in the brains of patients with AD. Several natural compounds have been shown to reduce oxidative stress in AD models through the Nrf2/HO-1 pathway (84, 85). We found that rapamycin reversed the reduced level of Nrf2/HO-1 in zinc-induced SH-SY5Y cells and rats, in line with previous observations (86). Rapamycin also rescued oxidative stress caused by tau hyperphosphorylation associated with inactive mTOR/P70S6K signaling pathway and active Nrf2/HO-1 signaling pathway.

Altered mTOR or P70S6K has been directly correlated with learning and memory in animal models (13, 87–89). Caccamo et al. (90, 91) found that inhibition of mTOR by rapamycin could improve learning and memory and reduce Aβ and tau pathology in 3×Tg-AD mice. Chronic treatment with rapamycin enhances learning and memory in young adult mice and improves age-related cognitive decline in older mice, possibly by activating major monoamine pathways in the brain (92). In the present study, we showed increased mTOR/P70S6K signaling in the hippocampus of rats. Rapamycin treatment rescued abnormal mTOR/P70S6K signaling and improved spatial learning in rats. Moreover, we showed that rapamycin treatment reversed the decreased expression levels of synaptic proteins SNAP 25, synaptophysin, and PSD-95 both in cell culture and in rats. Accumulating evidence has shown that rapamycin improves cognitive decline in a mouse model of Down syndrome (13, 93, 94), amyloidosis (J20) (95–98), and tauopathy (14, 99, 100). Given the coincident zinc accumulation and mTOR/P70S6K activation in damaged neurons in AD, we confirmed that pathological concentrations of zinc induced cell death and that rapamycin partially recued neuronal death in rats. Thus, rapamycin could exert an early intervention of zinc increases, and mTOR/P70S6K phosphorylation is a promising therapeutic strategy in the treatment of AD. Despite its compelling preclinical record, no clinical trials have tested rapamycin in patients with AD (101, 102).

Manczak and Reddy (103) found that mRNA expression levels were increased in synaptophysin (1.8-fold), synapsin 1 (2.2-fold), and synapsin 2 (2.0-fold) in siRNA-Tau cells relative to SHSY5Y cells, while the mRNA expression levels of synaptopodin (1.6-fold) and PSD-95 (1.3-fold) were increased but not significantly. We investigated whether tau phosphorylation is the major cause of presynaptic impairment through the generation of a tau knockout cell line. We showed that the protein levels of SNAP 25, synaptophysin, and PSD-95 in the tau siRNA of SH-SY5Y cells were not significantly increased (p = 0.248, p = 0.0071, p= 0.5105) (**Supplementary Figure 2**). These findings suggest that reduced tau is beneficial for maintaining synaptic function in SH-SY5Y cells, but the relation is not causal, since the absence of tau would be compensated by microtubule-associated protein 2 (MAP2) (104). Furthermore, Zhou et al. (105) showed that FTDP-17 mutant Tau mislocalized to presynaptic terminals in fly neurons, where it banded synaptic vesicles to elicit presynaptic dysfunction. Di et al. (106) found that in an inducible pseudophosphorylated tau (pathological human tau, PH-Tau) mouse model, low basal levels of PH-Tau (4% of endogenous tau) resulted in significant cognitive deficits, a decrease in the number of synapses in the CA1 region, a reduction in synaptic proteins, and localization to the nucleus.

There are several limitations in the study. First, only the MWM test was used to assess the spatial learning function of the rats. Further study using a panel of behavior tests will provide comprehensive insights into the effect of rapamycin on zinc-induced cognitive impairment. Second, we focused on the involvement of the mTOR/p70S6K and Nrf2/HO-1 pathways in the current study, while many other pathways have been implicated in the effect elicited by zinc and rapamycin. Moreover, the acute effects of zinc and rapamycin treatment were investigated in the current study. The effect of chronic or environmental zinc exposure and treatment using rapamycin *via* oral intake remains to be investigated. Advances in noninvasive imaging have enabled the detection of metal accumulation *in vivo* by using magnetic resonance imaging (107). A longitudinal study using *in vivo* imaging of the treatment effect on tau, neuroinflammation, and ROS in an animal model will provide further systematic insights (19, 108–110).

## Conclusions

In conclusion, zinc treatment induces mTOR/p70S6K activation and Nrf2/HO-1 inactivation, tau hyperphosphorylation, oxidative stress damage in SH-SY5Y cells and spatial learning impairment in rats. Rapamycin attenuated mTOR/p70S6k, increased Nrf2/HO-1 activity, and attenuated tau pathology, oxidative stress, and cognitive deficits induced by zinc in a rat model. Rapamycin might be a viable treatment for zinc-related neuronal and synaptic damage.

## Data Availability Statement

The original contributions presented in the study are included in the article/**Supplementary Material**. Further inquiries can be directed to the corresponding author.

## Ethics Statement

The animal study was reviewed and approved by Guiyang Regional Animal Care Center and Ethics Committee.

## Author Contributions

ZT contributed to the conception and study design. CL, YD, and QC performed the experiments. HL and SS contributed to data collection and data analysis. RN and ZT interpreted the data. CL, YD, RN, and ZT wrote the article. ZC performed the supplementary experiments during revision. All authors contributed to the article and approved the submitted version.

## Funding

This work was supported by the Chinese National Natural Science Foundation (81560241), China Postdoctoral Science Foundation (2020M683659XB), Foundation for Science and Technology projects in Guizhou ([2020]1Y354), Scientific Research Project of Guizhou Medical University (J[49]), and Scientific Research Project of Guizhou University of Traditional Chinese Medicine ([2019]48).

## Conflict of Interest

The authors declare that the research was conducted in the absence of any commercial or financial relationships that could be construed as a potential conflict of interest.

## Publisher’s Note

All claims expressed in this article are solely those of the authors and do not necessarily represent those of their affiliated organizations, or those of the publisher, the editors and the reviewers. Any product that may be evaluated in this article, or claim that may be made by its manufacturer, is not guaranteed or endorsed by the publisher.
