# Supplementary material for: Rapamycin Attenuated Zinc-Induced Tau Phosphorylation and Oxidative Stress in Rats: Involvement of Dual mTOR/p70S6K and Nrf2/HO-1 Pathways
Source: Front Immunol. 2022 Feb 7;13:782434. doi: 10.3389/fimmu.2022.782434 (PMC8858937; doi:10.3389/fimmu.2022.782434)
Supplement: Supplementary file 1 [file DataSheet_1.docx]

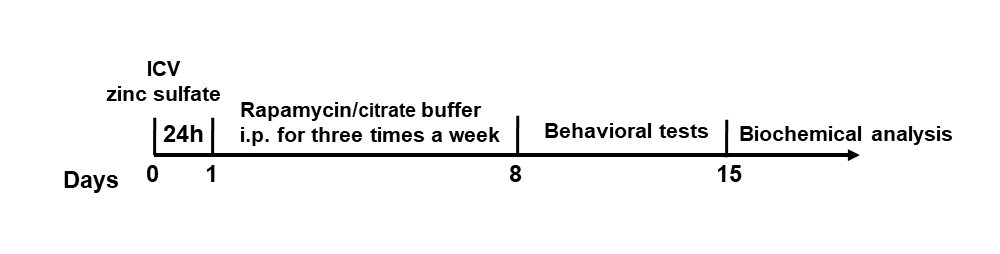


**Supplementary figure 1 Timeline of surgery, treatment and behavioral test in rats.** ICV: Intracerebroventricular

**
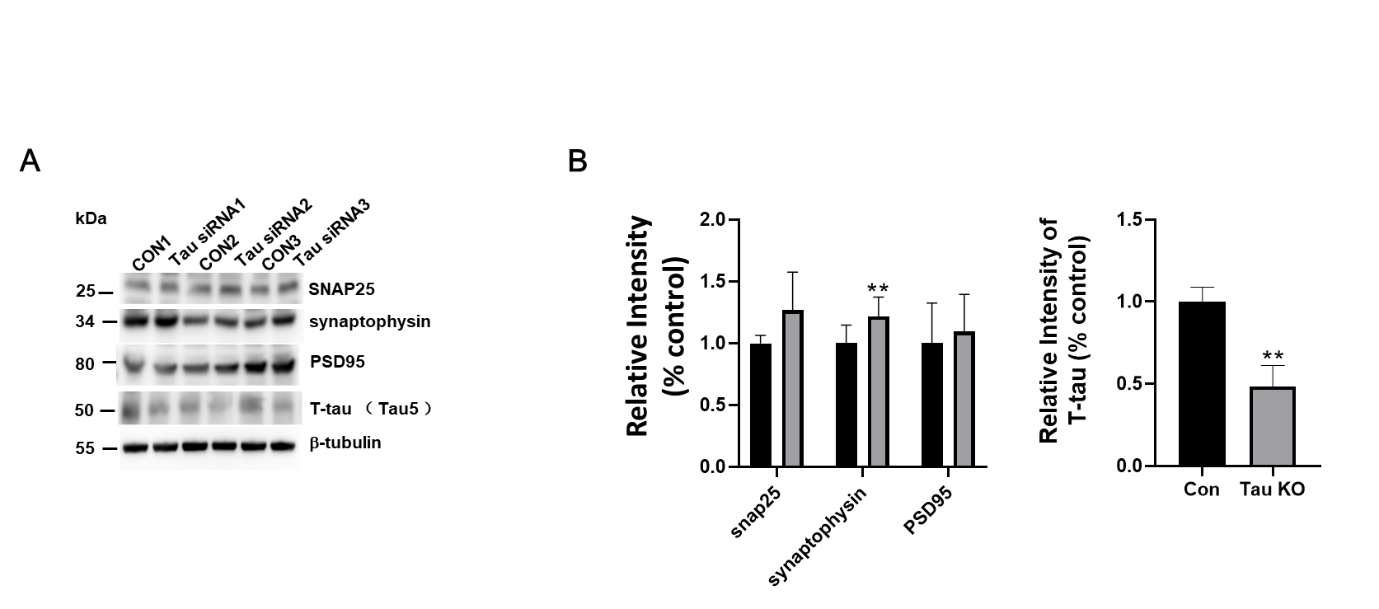
**

**Supplementary figure 2:** **Effects of tau deletion on the levels of synaptic proteins.** (A-B) Representative blots and quantification of the protein expression levels of SNAP25, synaptophysin, PSD95 and Total-tau in SH-SY5Y cells in the control and Tau siRNA groups. n = 3 cell experiments per group; **p < 0.01, ***p <0.001 vs. control group; Quantifications of the blots were normalized to β-tubulin.
